# Mouldable, thermoplastic, glue‐on frog‐supportive shoes change hoof kinetics in normal and obese Shetland ponies

**DOI:** 10.1111/evj.12814

**Published:** 2018-02-12

**Authors:** J. Sleutjens, F. M. Serra Bragança, M. W. van Empelen, R. E. ten Have, J. de Zwaan, E. Roelfsema, M. Oosterlinck, W. Back

**Affiliations:** ^1^ Department of Equine Sciences Faculty of Veterinary Medicine Utrecht University Utrecht the Netherlands; ^2^ Department of Surgery and Anaesthesia of Domestic Animals Faculty of Veterinary Medicine Ghent University Merelbeke Belgium

**Keywords:** horse, ponies, hoof kinetics, force and pressure plate, body condition score, subclinical laminitis, shoe

## Abstract

**Background:**

Obesity and hyperinsulinaemia are frequently encountered in the equine population and risk factors for the development of laminitis. There are many options for hoof support that claim a beneficial effect, but often the scientific evidence is scarce.

**Objectives:**

To quantify the effect of frog‐supportive shoes on hoof kinetics in normal and obese ponies.

**Study design:**

Controlled in vivo trial.

**Methods:**

Ten Shetland mares (n = 10) with a normal (n = 5) or obese (n = 5) body condition were led over a dynamically calibrated pressure plate before (T0), immediately after (T1) and 72 h (T2) after application of the shoes. The following locomotor variables were measured: stance duration (StDur), vertical impulse (VI), peak vertical force (PVF), time to PVF and time from PVF to lift off. The hoof print was divided into a toe and heel region and the StDur toe–heel index was calculated. The toe–heel hoof balance curves of the vertical force were plotted throughout the stance phase.

**Results:**

The VI and PVF increased significantly 72 h after application of the shoes, when compared with T0 and T1. The StDur toe–heel index and toe–heel balance curves were significantly different between the normal and obese ponies. These variables became more comparable between the groups after application of the frog‐supportive shoes.

**Main limitations:**

It would have been interesting to measure the effect of the shoe in patients with acute laminitis. However, this would have had major welfare implications.

**Conclusions:**

The obese ponies moved more carefully than the normal group, demonstrated by a decreased loading of the toe area. The data illustrate that the ponies became more comfortable 72 h after application of the shoes, with a pronounced effect in the obese group. Thus, these results suggest that frog‐supportive shoes could be beneficial, especially for obese ponies.

**Abbreviations**
BCS: Body condition score
BW: Body weight
EMS: Equine metabolic syndrome
Gluc_auc_: The area under the curve for glucose
I_auc_: The area under the curve for insulin
IQR: Interquartile range
PVF: Peak vertical force
StDur: Stance duration
TPVF to lift off: Time from peak vertical force to lift off
TPVF: Time to peak vertical force
VF: Vertical force
VI: Vertical impulse

## Introduction

Laminitis is a common condition in horses and ponies, with major welfare implications in the short‐ and long term. A study carried out in the UK, which was based on an owner questionnaire, reported that 15% of horses and ponies had a previous history of laminitis; of these, 46.2% were reported to have suffered from more than one episode, and the majority of patients (75.9%) with recurrent episodes were ponies 1. The prevalence of endocrine conditions such as pars pituitary intermedia dysfunction (PPID) and equine metabolic syndrome (EMS), potential underlying causes, was estimated to be as high as 89% in a group of 36 cases of laminitis, among which most patients were ponies 2. EMS is defined as a phenotype of obesity, insulin dysregulation and laminitis or a predisposition to developing laminitis 3. Obesity and hyperinsulinaemia are identified as risk factors for the development of pasture‐associated laminitis 4. Laminitis due to hyperinsulinaemia leads to specific lamellar pathology in natural occurring cases 5.

The current study was designed to measure in vivo the effect of a mouldable, thermoplastic, glue‐on, frog‐supportive shoe (Imprint shoe^®^)^a^. In this study, the effects of these shoes on hoof kinetics in normal and obese ponies were measured using a combined force and pressure plate system, which is a validated method to assess hoof balance and the clinical effect of different types of shoes 6, 7, 8, 9.

This study tested the hypothesis that there would be a significant difference in hoof kinetics (StDur, TPVF, PVF, TPVF to lift off, VI) between normal and obese ponies and that there would be an overall change in hoof kinetics after application of the shoes. Furthermore, it was hypothesised that the effect of the shoes on hoof kinetics would differ between the normal and obese ponies.

## Materials and methods

### Ponies

Shetland ponies from the research herd of the Faculty of Veterinary Medicine of Utrecht University were used for this study. The ponies were part of a larger study into the epigenetic effects of equine metabolic syndrome in embryos. The ponies that participated in the study were clinically sound, unshod for a period of at least 6 months and their feet were routinely trimmed every 6–8 weeks. The ponies had no clinical signs of current or previous laminitic episodes. The obese ponies (n = 5) had a mean (±s.d.) body mass of 244 ± 36 kg, age of 5 ± 1.8 years, height at the withers of 1.00 ± 0.05 m and a median (±IQR) body condition score (BCS) of 9.0 ± 0.75, using a validated scoring system with a scale from 1 to 10 10. The normal ponies (n = 5) had a mean (±s.d.) body mass of 185 ± 19 kg, age of 4 ± 1.5 years, height at the withers of 0.99 ± 0.02 m and a median (±IQR) BCS of 6.0 ± 1.75 10.

### Oral glucose tolerance test

Before the start of this study, an oral glucose tolerance test was performed 11. The ponies were starved during 12 h. A jugular catheter was placed, after disinfecting and locally anaesthetising the injection area with 2% lidocaine. Next, a nasogastric tube was placed and 1.0 g/kg BW glucose, dissolved in 2 L water, was given through the tube within 2 min. Blood samples (8 mL) were collected at t = 0, 30, 60, 90, 120, 180, 240 and 300 min after administration of glucose. Glucose concentrations were measured in all blood samples using an enzymatic spectrophotometer^b^, serum insulin concentrations were measured only in samples taken at t = 0, 30, 60, 90 and 120 min, using a new commercially available solid‐phase, enzyme‐labelled chemiluminiscent immunometric assay^c^ (CLIA – Immulite 2000 Insulin) designed for human insulin. The area under the curves for glucose (Gluc_auc_) and insulin (I_auc_) were calculated by integration (using a linear trapezoidal method) and compared between the normal and obese ponies.

### Application of the mouldable, thermoplastic, frog‐supportive shoes

**Figure 1:**
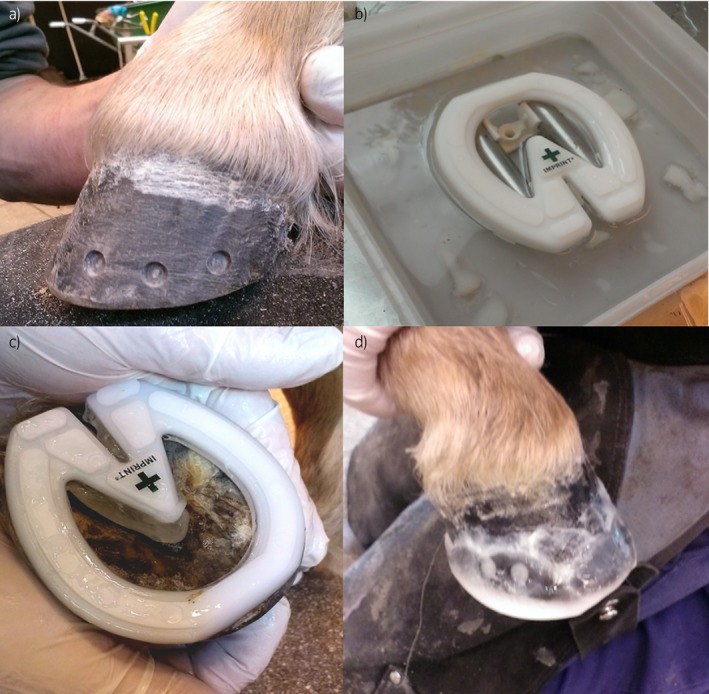
Schematic illustration that summarises the application process of the frog‐supportive mouldable shoes: a) Six superficial indentations are drilled in the dorsal hoof wall to increase the contact area between the hoof wall and the mouldable plastic. This is followed by the application of an adhesive glue to the hoof wall. b) The upper rim of the shoe becomes mouldable after it has been emerged in boiling water. c) The correct size frog‐supportive shoe is fitted on the foot. d) The mouldable thermoplastic hardens out within minutes with the application of a freezer spray.

The ponies were studied on the combined force and pressure plate system immediately after routine foot trimming (maintaining a straight hoof axis, conserving the frog and trimming the hoof wall as needed) (T0). After the first measurements had been taken (as described below), Imprint^®^ shoes were applied to both front feet as instructed by the manufacturer^a^ (Fig 1) and immediately after application, the measurements were repeated (T = 1). Finally, the measurements were repeated 72 h after application of the shoes (T = 2). The trimming, as well as the application of the shoes, was performed by a single experienced farrier (J.d.Z.). The study was approved by the animal ethical committee of Utrecht University.

### Data collection

After a 5‐min warming‐up period, which consisted of walking in‐hand in straight lines, the ponies were led over the measuring system, first at the walk and subsequently at the trot. The measuring system consisted of a pressure plate (Footscan 3D 1 m system)^d^ with a spatial resolution of 2.6 sensors/cm^2^, and a pressure range of 0–200 N/cm^2^, mounted on top of a force platform (Z4852C)^e^ in the middle of a 20‐m long track, covered with a 5‐mm thick rubber mat with a shore hardness of 65 ± 5 (NR/SBR)^f^, as used in previous studies 7, 8, 12. The force and pressure plate were sampling at 250 Hz and recording was triggered at contact with the force/pressure plate. The pressure plate was dynamically calibrated by the force plate. Two pairs of photoelectric sensors (WE260‐S270)^g^ that were positioned 2 m apart, perpendicular to the track were used to record the average speed. Although acceleration was not measured directly, the length of the track ensured that the effect of acceleration and deceleration at the start and end of each trial was minimised over the central measuring area. Five valid measurements were collected for each front hoof at both the walk and trot. A trial was considered valid if the pony looked straight ahead and maintained a constant pace over the measuring system, if a complete print of at least one front hoof was recorded, and if the speed was within a preset range of 0.8–1.4 m/s at the walk and 2.5–3.5 m/s at the trot 12.

### Data processing

**Figure 2:**
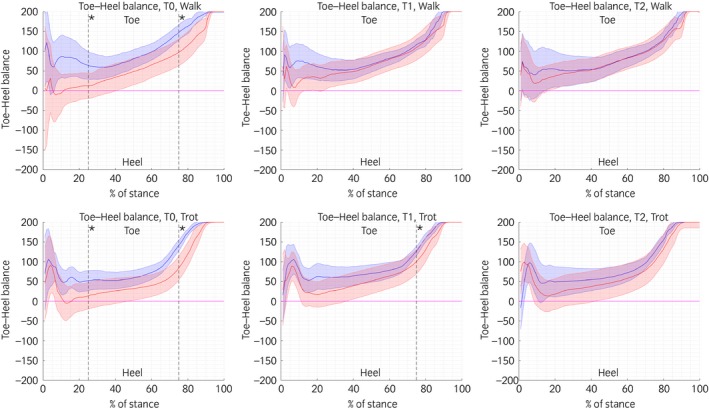
Schematic representation of the median toe–heel balance curves at the walk and trot: in blue the median ± median absolute deviation (mad) of the normal ponies, and in red the median ± mad of the obese ponies. * = Significant difference P<0.017 after Bonferonni correction compared with the normal group. An index of 0 indicates perfect balance in vertical ground reaction forces between the two regions, whereas positive and negative values indicate relatively higher loading of the toe and heel region, respectively.

The mean from five valid trials of each of the following parameters was calculated for the right and left front hoof; this was done for each individual pony at both walk and trot. Therefore, at each time point, 20 measurements (5 ponies; left and right hoof; walk and trot) were collected for the normal and obese group. For each front hoof, the following variables were calculated at the walk and the trot: stance duration (StDur), expressed in milliseconds (ms); vertical impulse (VI), calculated by time integration of the force–time curves (N s) and normalised for individual body mass (kg); peak vertical force (PVF), calculated as the maximal vertical force (N) normalised for individual body mass (kg); time to peak vertical force (TPVF), which is the time at which the maximal force occurred (ms); and time from peak vertical force to lift off (TPVF to lift off) (ms) 8, 12. Data are presented as mean ± s.d. All temporal variables were calculated from the pressure plate; the force plate was used to calibrate the force data (PVF and VI) recorded by the pressure plate. Each individual hoof print was divided visually into a toe and a heel region by a line through the maximal hoof width, as described previously 7. Subsequently, the StDur toe–heel index was calculated as [(StDur_Toe_−StDur_Heel_)/0.5(StDur_Toe_ + StDur_Heel_)] * 100% and presented as median ± interquartile range (IQR). The toe–heel balance was calculated as [(VF_Toe_−VF_Heel_)/0.5(VF_Toe_ + VF_Heel_)] * 100% 7. Using this method, an index of 0 indicates equal contribution of the toe and heel region for the measured variable, whereas positive or negative values (range 200 to −200%) indicate a relatively higher contribution of the toe or heel region, respectively. The toe–heel balance data are presented as median ± median absolute deviation (mad) curves throughout the stance phase (Fig 2) 7.

### Data analysis

Custom‐made Matlab scripts (Matlab R2015a)^h^ were used for processing the data and IBM SPSS Statistics 22^i^ for the statistical analysis. A Shapiro–Wilk test and Levene's test were performed on the variables Gluc_auc_ and I_auc_ to assure the assumptions of normality and equal variances. A natural log transformation was applied to I_auc_ in order to meet the assumption of normality. An independent *t*‐test was used to calculate the difference in Gluc_auc_ and I_auc_ between both groups. The hoof kinetic variables were checked for normality by the Shapiro–Wilk test and normal distribution of the residuals was verified using Q–Q plots. A natural log transformation was applied to the variable TPVF in trot in order to meet the assumption of normality. The variables TPVF, PVF, TPVF to lift off, StDur, VI and speed were tested with a linear mixed model. The model was constructed with ‘pony’ as a random effect, time point (T0, T1, T2), BCS, limb, and interaction between time point and BCS were tested as fixed factors. The best model was chosen on the basis of the lowest Akaike information criterion (AIC) score. Pairwise comparisons were performed and a post hoc Bonferonni correction was applied by dividing the alfa of P<0.05 by the number of pairwise comparisons. The StDur toe–heel index and the toe–heel balance at five selected time points (1, 25, 50, 75 and 100% of the stance phase) were not distributed normally. Consequently, independent nonparametric Kruskal–Wallis tests or Mann–Whitney *U* tests were applied with pairwise comparisons. A post hoc Bonferroni correction was applied by dividing the alpha of P<0.05 by the number of pairwise comparisons. A P≤0.05 was used to indicate statistical significance unless stated otherwise.

## Results

### Oral glucose tolerance test

The Gluc_auc_ mmol min/L (mean ± s.d.) was 2259 ± 599 in the normal and 1725 ± 214 in the obese group. The I_auc_ μU min/mL (mean ± s.d.) was 584 ± 400 in the normal group and 6219 ± 8043 in the obese group (P<0.03).

### Effect of limb

There was no significant difference between the left and right hoof, except for the variable PVF to lift off in walk. Therefore, the measured variables from both hooves were pooled, and the difference between the left and right hoof for PVF to lift off is mentioned in the appropriate section.

### Effect of BCS

The differences between the normal and obese group were analysed irrespective of time point. Therefore, for each group, 60 measurements were included (5 ponies; 3 time points; left and right hoof; walk and trot). At the walk, the StDur (ms) was 675.6 ± 41.6 in the normal and 582.7 ± 51.4 (P<0.002) in the obese group. The StDur toe–heel index was 13.5 ± 6.3 in the normal and 9.2 ± 2.9 (P<0.001) in the obese group, which means that the heel region contributed relatively more than the toe region to the total StDur in the obese group. The VI (N s/kg) was 2.6 ± 0.4 in the normal and 2.0 ± 0.5 (P<0.017) in the obese group. The time from PVF to lift off (ms) was 230 ± 12.4 in the normal and 194 ± 17.9 (P<0.001) in the obese group. The other variables did not differ significantly between the BCS groups.

At the trot, the PVF (N/kg) was 9.5 ± 1.8 in the normal and 7.8 ± 1.4 in the obese group (P<0.02). The StDur toe–heel index was 16.6 ± 4.9 in the normal and 11.6 ± 2.5 (P<0.001) in the obese group. The other variables did not differ significantly between the BCS groups (Table 1).

**Table 1:** The effect of body condition score on hoof kinetics in the walk and trot. The data are presented as mean ± s.d., the StDur toe–heel index is presented as median ± IQR

	BCS	TPVF (ms)	PVF (N/kg)	TPVF lift off (ms)	StDur (ms)	VI (N s/kg)	StDur toe–heel index	Speed (m/s)
Walk	Normal	430 ± 37	5.7 ± 1.0	230 ± 12.4	675.6 ± 41.6	2.6 ± 0.4	13.5 ± 6.3	1.1 ± 0.1
	Obese	399 ± 36	5.1 ± 1.0	194 ± 17.9^a^	582.7 ± 51.4^a^	2.0 ± 0.5^a^	9.2 ± 2.9^a^	1.2 ± 0.1
Trot	Normal	126 ± 22	9.5 ± 1.8	165 ± 33.2	239.6 ± 24.9	1.3 ± 0.3	16.6 ± 4.9	2.8 ± 0.1
	Obese	126 ± 19	7.8 ± 1.4^a^	138 ± 28.8	226.0 ± 30.5	1.1 ± 0.3	11.6 ± 2.5^a^	2.8 ± 0.2

a Significant difference P<0.05 compared with the normal group.

### Effect of the mouldable, thermoplastic, frog‐supportive shoes

After Bonferonni correction, a P<0.017 was used to indicate statistical significance. The differences between time points were analysed irrespective of BCS. Therefore, at each time point, 40 measurements were included (10 ponies; left and right hoof; walk and trot). At the walk, the VI (N s/kg) decreased from T0 (2.3 ± 0.5) to T1 (2.0 ± 0.4) and increased again at T2 (2.6 ± 0.5); the differences were significant between T0 and the subsequent time points (P<0.002) and between T1 and T2 (P<0.001). The PVF (N/kg) decreased from T0 (5.4 ± 1.0) to T1 (4.8 ± 0.9) and increased at T2 (6.1 ± 0.9); the difference between T0 and T2 (P<0.003) and between T1 and T2 was significant (P<0.001). The other variables did not change significantly between time points.

At the trot, the VI (N s/kg) was increased at T2 (1.3 ± 0.2) compared with T0 (1.1 ± 0.3) and T1 (1.1 ± 0.3); the differences between T2 and the previous time points were significant (P<0.001). The PVF (N/kg) was increased at T2 (9.8 ± 1.6) compared with T0 (8.1 ± 1.7) and T1 (8.0 ± 1.7); the differences between T2 and the previous time points were significant (P<0.001). The other variables did not change significantly between time points (Table 2).

**Table 2:** The effect of the frog‐supportive shoes on hoof kinetics in the walk and trot T0 = the reference (control) value, T1 = immediately after application of the frog‐supportive shoes, T2 = 72 h after application of the frog‐supportive shoe. The data are presented as mean ± s.d., the StDur toe–heel index is presented as median ± IQR

	Time	TPVF (ms)	PVF (N/kg)	TPVF to lift off (ms)	StDur (ms)	VI (N s/kg)	StDur toe–heel index	Speed (m/s)
Walk	0	410 ± 39	5.4 ± 1.0	212. ± 25.1	629.5 ± 73.2	2.3 ± 0.5	10.8 ± 5.8	1.2 ± 0.1
	1	416 ± 35	4.8 ± 0.9	211 ± 24.4	629.8 ± 64.8	2.0 ± 0.4^a^	10.8 ± 5.7	1.2 ± 0.1
	2	417 ± 46	6.1 ± 0.9^a^^,^ b	213 ± 22.5	628.1 ± 62.5	2.6 ± 0.5^a^^,^ b	9.6 ± 4.7	1.2 ± 0.0
Trot	0	128 ± 22	8.1 ± 1.7	151 ± 35.6	235.1 ± 30.9	1.1 ± 0.3	14.0 ± 5.5	2.8 ± 0.1
	1	127 ± 19	8.0 ± 1.7	152 ± 33.5	235.3 ± 25.8	1.1 ± 0.3	12.3 ± 5.3	2.8 ± 0.1
	2	122 ± 20	9.8 ± 1.6^a^^,^ b	151 ± 33.6	228.0 ± 29.3	1.3 ± 0.2^a^^,^ b	13.6 ± 5.4	2.9 ± 0.1

a Significant difference P<0.017 after Bonferonni correction compared with T0.

b Significant difference P<0.017 after Bonferonni correction compared with T1.

### Interaction between BCS and the frog‐supportive shoes

After Bonferonni correction, a P<0.017 was used to indicate statistical significance. The differences between the normal and obese ponies were analysed at each time point separately. Therefore, 20 measurements were included (5 ponies; left and right hoof; walk and trot) for each BCS group at separate time points. At the walk, the StDur (ms) was higher in the normal than in the obese group at T0 (690.2 ± 35.9 vs. 568.7 ± 42.7; P<0.001), at T1 (681.2 ± 22.3 vs. 578.5 ± 50.1; P<0.001), and at T2 (655.2 ± 55.2 vs. 601.0 ± 59.7), but not significantly. The TPVF to lift off (ms) was longer in the normal than in the obese group at T0 (232 ± 10.2 vs. 192 ± 19.0; P<0.001), at T1 (230 ± 9.8 vs. 191 ± 17.6; P<0.001), and at T2 (228 ± 16.8 vs. 199 ± 18.1; P<0.001). There was a significant difference between limbs (P<0.02). In the left hoof, the TPVF to lift off (ms) was longer in the normal than in the obese group at T0 (233 ± 11.3 vs. 197 ± 17.4; P<0.002), at T1 (235 ± 8.5 vs. 197 ± 18.9; P<0.001) and at T2 (225 ± 14.9 vs. 205 ± 19.6), but not significantly. In the right hoof, the TPVF to lift off (ms) was longer in the normal than in the obese group at T0 (231 ± 10.1 vs. 188 ± 21.3; P<0.001), at T1 (225 ± 8.9 vs. 185 ± 15.4; P<0.001) and at T2 (230 ± 19.8 vs. 192 ± 15.4; P<0.001). The StDur toe–heel index was also higher in the normal as compared with the obese group at T0 (14.2 ± 5.8 vs. 9.4 ± 3.6; P<0.002), at T1 (14.2 ± 6.4 vs. 9.5 ± 2.4; P<0.002), and at T2 (12.5 ± 7.9 vs. 9.1 ± 2.7; P<0.011). The speed (m/s) of the obese ponies at T0 (1.2 ± 0.1) was slightly higher than that of the normal ponies (1.1 ± 0.1; P<0.01). Other variables did not differ significantly between the groups at different time points.

At the trot, the PVF (N/kg) was higher in the normal as compared with the obese group at all time points, but only significantly at T2 (11.1 ± 1.1 vs. 8.5 ± 0.7; P<0.002). The StDur toe–heel index was higher in the normal than in the obese group at T0 (17.1 ± 4.4 vs. 11.8 ± 2.1; P<0.001) and at T1 (15.9 ± 6.5 vs 11.2 ± 1.9; P<0.015). The other variables did not differ significantly between the groups at different time points (Table 3).

**Table 3:** The effect of the interaction between body condition score and the frog‐supportive shoes on hoof kinetics in the walk and trot T0 = the reference (control) value, T1 = immediately after application of the frog‐supportive shoes, T2 = 72 h after application of the frog‐supportive shoe. The data are presented as mean ± s.d., the StDur toe–heel index is presented as median ± IQR

	T	BCS	TPVF (ms)	PVF (N/kg)	TPVF to lift off (ms)	StDur (ms)	VI (N s/kg)	StD toe–heel index	Speed (m/s)
Walk	0	Normal	435 ± 26	5.4 ± 1.0	232 ± 10.2	690.2 ± 35.9	2.5 ± 0.5	14.2 ± 5.8	1.1 ± 0.1
	0	Obese	386 ± 35	5.3 ± 1.0	192 ± 19.0^a^	568.7 ± 42.7^a^	2.1 ± 0.5	9.4 ± 3.6^a^	1.2 ± 0.1^a^
	1	Normal	435 ± 29	5.2 ± 0.7	230 ± 9.8	681.2 ± 22.3	2.3 ± 0.3	14.2 ± 6.4	1.1 ± 0.1
	1	Obese	398 ± 33	4.5 ± 0.9	191 ± 17.6^a^	578.5 ± 50.1^a^	1.7 ± 0.4	9.5 ± 2.4^a^	1.2 ± 0.1
	2	Normal	422 ± 53	6.6 ± 0.8	228 ± 16.8	655.2 ± 55.2	2.9 ± 0.3	12.5 ± 7.9	1.2 ± 0.1
	2	Obese	412 ± 39	5.6 ± 0.8	199 ± 18.1^a^	601.0 ± 59.7	2.3 ± 0.5	9.1 ± 2.7^a^	1.2 ± 0.0
Trot	0	Normal	125 ± 21	8.6 ± 1.7	165 ± 34.8	240.4 ± 23.3	1.2 ± 0.3	17.1 ± 4.4	2.8 ± 0.2
	0	Obese	131 ± 25	7.5 ± 1.7	136 ± 31.7	229.8 ± 37.6	1.0 ± 0.3	11.8 ± 2.1^a^	2.9 ± 0.1
	1	Normal	130 ± 21	8.8 ± 1.5	165 ± 35.2	243.9 ± 21.6	1.2 ± 0.3	15.9 ± 6.5	2.8 ± 0.1
	1	Obese	125 ± 17	7.3 ± 1.6	140 ± 28.0	226.6 ± 27.8	1.0 ± 0.3	11.2 ± 1.9^a^	2.8 ± 0.1
	2	Normal	123 ± 25	11.1 ± 1.1	164 ± 33.3	234.5 ± 30.7	1.5 ± 0.1	15.6 ± 6.8	2.9 ± 0.1
	2	Obese	122 ± 14	8.5 ± 0.7^a^	138 ± 29.8	221.5 ± 27.9	1.1 ± 0.2	12.0 ± 3.0	2.9 ± 0.1

a Significant difference P<0.017 after Bonferonni correction compared with the normal group.

### Hoof balance curves

After Bonferonni correction, a P<0.017 was used to indicate statistical significance. At the walk, overall the VF toe–heel index was significantly higher in the normal than in the obese group at 25% (P<0.019) and 75% (P<0.004) of the stance phase. At T0, the VF toe–heel index was significantly higher in the normal than in the obese group at 25% (P<0.009) and 75% (P<0.002) of the stance phase. There was no significant difference between the groups at T1 and T2.

At the trot, overall the VF toe–heel index was significantly higher in the normal than in the obese group at 25% (P<0.002), 50% (P<0.014), and 75% (P<0.001) of the stance phase. At T0, the VF toe–heel index was significantly higher in the normal than in the obese group at 25% (P<0.007) and 75% (P<0.001) of the stance phase. At T1, the VF toe–heel index was significantly higher in the normal than in the obese group at 75% (P<0.009) of the stance phase. There was no significant difference between the groups at T2 (Fig 2).

## Discussion

The current study demonstrated a biomechanical effect of the frog‐supportive shoes and an overall difference in hoof kinetic variables between normal and obese, hyperinsulinaemic ponies. This is the first study to demonstrate a significant effect of BCS on hoof kinetics. This finding is clinically relevant because horses with access to pasture for ≥6 h daily had a 35.4% prevalence of a BCS≥7/9 during summer and 27.08% at the end of winter 13. These numbers are comparable with the prevalence of obesity reported by studies based on owner questionnaires 14. At the walk and trot, the hoof balance curves of the obese ponies showed increased loading of the heel region as compared with the toe region (Fig 2). Furthermore, the StDur toe–heel index demonstrated that the heel region contributed relatively more to the total stance time than the toe region. At the walk, the total StDur and the TPVF to lift off, which could also be described as the time of breakover, were significantly lower in the obese group than in the normal group, without a significant difference in speed between the BCS groups (Table 1). The combination of these findings could indicate that the obese ponies experienced some degree of discomfort or pain in their toe region, possibly because they were in a subclinical stage of laminitis, given that obesity and hyperinsulinaemia are risk factors for the development of laminitis 4.

Important aspects in the treatment of (acute) laminitis are to support the feet in order to 1) reduce stress on the dorsal hoof wall, 2) reduce pressure on the sensitive area of the sole immediately below the dorsal margin of the distal phalanx and 3) in some cases to decrease the tension exerted by the deep digital flexor tendon 15. This can be accomplished using foam sole support, which in healthy horses has been shown to increase the total foot contact surface, decrease the total foot contact pressure as well as peak foot contact pressure and position the centre of pressure more palmarly after 48 h 16. A reduction in stress on the dorsal hoof wall can be achieved by recruiting the palmar structures of the hoof, including the frog, into the weightbearing surface, as shown in vitro by the application of a hoof cast with heel wedge and a therapeutic shoe with an open toe and palmar bar 17. Similarly, an in vivo study demonstrated that the peak strain in the deep digital flexor tendon is reduced by the application of an egg‐bar shoe and a shoe with heel wedge 18. Finally, the use of a rolled‐toe shoe may be considered, as this shoe causes a more gradual and smooth hoof‐enrolment pattern but it does not reduce the total stance time or breakover duration 19.

The frog‐supportive shoes tested in the current study have been developed to support the feet in patients with acute laminitis. The proposed theory behind the shoe is that, owing to the heart bar shape, the palmar structures of the hoof contribute to the weightbearing surface and the rolled toe should ease breakover 19. In the present study, we compared the effect of the shoes between normal and obese ponies with hyperinsulinaemia. This study demonstrated that several variables of hoof kinetics, mainly the StDur toe–heel index and the hoof balance curves, became more similar between normal and obese ponies after the application of the frog‐supportive shoes. Compared with the hoof support applied in the aforementioned studies 17, 18, 19, no nailing is required with the application of the thermoplastic frog‐supportive shoes. Furthermore, the studied shoes can stay on for several weeks, compared with the foam support which needs to be replaced more frequently 16.

Overall, the frog‐supportive shoes mainly affected VI and PVF at the walk and trot. Without a significant change in StDur, it is logical that a difference in PVF is followed by a comparable change in VI. Both variables demonstrated a significant increase at T2 compared with the previous time points. Based on an earlier study in which PVF increased in lame horses, treated with a nonsteroidal anti‐inflammatory drug resulting in improved lameness scores 20, the measured increase in PVF in the current study could suggest that overall the ponies became more comfortable after application of the frog‐supportive shoes.

In conclusion, the increase in PVF and VI 72 h after the application of the frog‐supportive shoes, and the more comparable StDur toe–heel index and toe–heel balance curves between normal and obese ponies after application of the shoes, would suggest a beneficial effect of the shoe in normal and especially in obese, hyperinsulinaemic ponies.

## Authors’ declaration of interests

None of the authors of this paper has a financial or personal relationship with other people or organisations that could inappropriately influence or bias the content of the paper. The authors are grateful to Andrew Poynton (http://www.imprintshoes.co.uk/) for providing the required shoes and materials.

## Ethical animal research

The study was approved by the Ethical Committee of Utrecht University (approval number DEC 2014. III.02.021).

## Sources of funding

None.

## Author contributions

J. Sleutjens contributed to study design, data analysis and interpretation, and preparation of the manuscript. F.M. Serra Bragança contributed to study design, data analysis and interpretation, and preparation of the manuscript. W.M van Empelen contributed to study design, study execution, initial data analysis and interpretation. R.E. ten Have contributed to study design, study execution, initial data analysis and interpretation. J. de Zwaan contributed to study execution. E. Roelfsema contributed to execution and data analysis of the oral glucose tolerance test, and preparation of the manuscript. M. Oosterlinck contributed to study design and preparation of the manuscript. W. Back contributed to study design and preparation of the manuscript. All authors gave their final approval of the manuscript.

## Manufacturers' addresses

^a^Imprint Equine foot care, Poynton Ltd, Malmesbury, Wiltshire, UK. (http://www.imprintshoes.co.uk/)

^b^Beckman Coulter Nederland B.V., Woerden, The Netherlands.

^c^Siemens Diagnostic Products Corp, Los Angeles, California, USA.

^d^RSscan International NV, Paal, Belgium.

^e^Kistler, Winterthur, Switzerland.

^f^De Mulder Rubber and Plastics, Gent, Belgium.

^g^Sick AG, Düsseldorf, Germany.

^h^MathWorks, Natick, Massachusetts, USA.

^i^IBM Corporation, New York, USA.
